# Investigation of the Impact of Cross-Polymerization on the Structural and Frictional Properties of Alkylsilane Monolayers Using Molecular Simulation

**DOI:** 10.3390/nano9040639

**Published:** 2019-04-19

**Authors:** Jana E. Black, Andrew Z. Summers, Christopher R. Iacovella, Peter T. Cummings, Clare McCabe

**Affiliations:** 1 Department of Chemical and Biomolecular Engineering, Vanderbilt University, Nashville, TN 37235, USA; jana.black@vanderbilt.edu (J.E.B.); andrew.z.summers@vanderbilt.edu (A.Z.S.); christopher.r.iacovella@vanderbilt.edu (C.R.I.); peter.cummings@vanderbilt.edu (P.T.C.); 2 Department of Chemical and Biomolecular Engineering and Department of Chemistry, Vanderbilt University, Nashville, TN 37235, USA

**Keywords:** molecular dynamics, tribology, surface science, self-assembled monolayers, cross-polymerization, adsorption, chemisorption, physisorption

## Abstract

Cross-linked chemisorbed *n*-alkylsilane (CH_3_(CH_2_)*_n_*_−1_Si(OH)_3_) monolayers on amorphous silica surfaces have been studied and their structural properties and frictional performance were compared to those of equivalent monolayers without cross-linkages. The simulations isolated for the first time the effects of both siloxane cross-linkages and the fraction of chains chemisorbed to the surface, providing insight into a longstanding fundamental question in the literature regarding molecular-level structure. The results demonstrate that both cross-linkages and the fraction of chemisorbed chains affect monolayer structure in small but measurable ways, particularly for monolayers constructed from short chains; however, these changes do not appear to have a significant impact on frictional performance.

## 1. Introduction

Micro- and nanoelectromechanical systems (MEMSs and NEMSs) have been used to develop smaller and more efficient sensors to detect chemical signals, stresses, vibrations, and forces at the atomic level [1,2]; examples include tips and cantilever beams in atomic force microscopy [3] and inertial navigation system accelerometers and gyroscopes [4]. MEMS/NEMS devices have small lateral dimensions and therefore large surface-area-to-volume ratios, which can result in significant surface interactions (e.g., adhesion and friction) that can lead to surface damage and eventual device failure [2,5,6]. An effective method to protect and lubricate contacting surfaces in such devices is to employ chemisorbed or physisorbed monolayers, which provide dense, two-dimensional sheets of surface-bound films that modify interfacial properties and reduce the risk of direct contact between surfaces [7,8,9,10,11,12]. The tribological properties of monolayer-coated surfaces primarily depend on the structure of the monolayer itself, which can be tailored by manipulating the composition of the precursor molecules (i.e., monomers) and/or the structure of the underlying surface [13,14,15,16]. Different types of monolayers assembled on a wide variety of surfaces have been shown to reduce static friction (i.e., stiction) and surface damage due to oxidation and wear [9,10,11,12].

Many MEMS/NEMS devices are fabricated from oxidized silicon (SiO_2_) [11,17,18], and interest in lubrication schemes for such devices has led to numerous studies on the tribological behavior of organosilane monolayers, as they can bond to oxidized surfaces [17,19,20,21,22,23,24,25,26,27]. The most commonly used organosilane molecules are monoalkylsilanes (RSiX_3_, where “R” is a linear alkyl group and “X” is a hydrolyzable leaving group) [28]. The hydrolysis rate of “X” is known to play a significant role in both the formation and final structure of the monolayers. For example, Naik et al. compared monolayers constructed from octadecylsilane molecules with chloro, methoxy, and ethoxy leaving groups (i.e., CH_3_(CH_2_)_16_CH_2_–SiX_3_, where “X” is Cl, OCH_3_, or OCH_2_CH_3_); the trichloroalkylsilanes rapidly formed a densely packed, highly organized monolayer, whereas the other two monolayers remained sparse and disorganized following much longer immersion times [29]. The difference was primarily attributed to the much faster hydrolysis rate of Cl, as compared with methoxy and ethoxy groups. Note that high packing densities are crucial to the tribological performance of alkylsilane monolayers, and so they are generally prepared using trichloroalkylsilane monomers [9,17,19,20,21].

**Figure 1:**
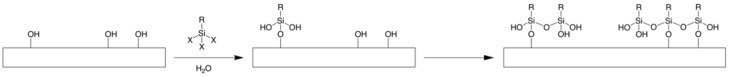
Proposed mechanism by which monoalkylsilanes (RSiX_3_, where “R” is a linear alkyl group and “X” is a hydrolyzable leaving group) are adsorbed onto oxidized surfaces (e.g., silica, titania, and alumina). Although described sequentially, two or more of these steps may occur through a concerted mechanism as described in Ref. [28].

Although the exact mechanism by which alkylsilanes are adsorbed onto oxidized surfaces remains unclear, the most widely accepted model is illustrated in Figure 1 [28]. In the presence of water, alkylsilanes are hydrolyzed to form alkylsilanols, which react with each other to form Si–O–Si (siloxane) linkages and also with surface-bound hydroxyl groups via condensation reactions that release water. Although described sequentially, two or more of these steps may occur through a concerted mechanism [28]. Monolayer formation is thought to follow an “island growth” mechanism, whereby the first monomers that bond to the surface, the mobility of which is limited by surface attachment and cross-polymerization to neighboring monomers, serve as nucleation sites for the remainder of the monolayer to form [30,31]. This classical model of the reaction mechanism raises two fundamental questions regarding alkylsilane monolayer structure: to what degree do alkylsilanes (1) cross-polymerize via siloxane linkages and (2) form covalent bonds to surface-bound hydroxyl groups?

Alkylsilane monolayer stability and robustness are generally attributed to cross-polymerization via the siloxane linkages [30,32,33,34,35,36,37,38,39]. The degree to which alkylsilanes cross-polymerize has been previously studied, but the results are difficult to interpret because they appear to pose conflicting requirements on monolayer structure. Data obtained using several different methods (e.g., X-ray photoelectron spectroscopy [39], X-ray scattering [40], nuclear magnetic resonance [41], and infrared spectroscopy [29,40,41,42,43,44,45]) suggest that extensive cross-polymerization occurs; it is estimated that each monomer forms an average of ~1.5–2 siloxane linkages to other monomers [41]. The length of Si–O siloxane bonds varies between ~1.59 and 1.65 Å, so the largest possible distance between two Si atoms connected via a siloxane linkage is ~3.3 Å [40,46,47]. However, the average distance between neighboring monomers in densely packed alkylsilane monolayers is observed to be ~4.5 Å [47]. Note that parallel linear alkanes must be separated by a minimum distance of ~4.2 Å due to steric hindrance [48], so only minimal tilting/bending of the alkyl “R” groups can occur in dense monolayers. Under these conditions, cross-polymerization via siloxane linkages would be limited to a few small alkylsilane oligomers (two to six monomers) [40]. This apparent discrepancy could be explained if alkylsilane monolayers exist in a state of dynamic equilibrium involving the rapid breakage and reformation of siloxane Si–O bonds; at any instant, the monolayer would consist of monomers and small oligomers with the continuous redistribution of siloxane linkages creating the overall effect of an extensively cross-polymerized monolayer [40,49]. This idea is supported by the unusual observation that alkylsilane polar head groups are highly mobile about their equilibrium in-plane positions, while the alkyl “R” groups form a stable configuration [40]. Plueddemann asserted that continuous Si–O bond breakage and reformation proceed via hydrolysis/condensation reactions (i.e., the addition/loss of water molecules) [49]; note that a submonolayer amount of water is expected to be physisorbed via hydrogen bonds with alkylsilanol and surface-bound hydroxyl groups [42]. Maoz et al. later proposed an alternate mechanism, where Si–O bonds are continuously redistributed among Si–O–Si (siloxane) and Si–O–H (silanol) groups; in this case, the activation energy required for Si–O bond breakage is thought to be supplied by simultaneous Si–O bond formation [40].

Alkylsilanes may be hydrogen bonded (physisorbed) or covalently bonded (chemisorbed) to the surface via surface-bound hydroxyl groups. Some experimental studies suggest these reactions compete near oxidized surfaces; if the density of surface-bound hydroxyl groups is high, monomers will readily form covalent bonds to the surface, but otherwise, they are more likely to form cross-linkages with other monomers and/or hydrogen bonds with surface oxygen atoms [28,39,43,44,47]. It is difficult to measure the fraction of monomers that become chemisorbed and is even more difficult to control due to the large number of variables involved as well as challenges related to understanding/regulating reactions with water molecules near the surface [37,39,50]. Some of the variables involved include alkylsilane structure (e.g., reactivity of hydrolyzable “X” groups) [28,45], surface structure (e.g., density of surface-bound hydroxyl groups) [28,39], and conditions during monolayer formation (e.g., temperature [41] and amount of water present [28,39]). The fraction of chemisorbed chains is expected to play a role in monolayer structure and durability [37,39,50]. For example, Allara et al. compared octadecylsiloxane monolayers on inert gold substrates to those on oxidized silicon (i.e., SiO_2_) featuring a high density of surface-bound hydroxyl groups (~5 OH/nm^2^), which are capable of reacting with hydrolyzed alkylsilane monomers. They found monolayers with few or no covalent bonds to the SiO_2_ surfaces to be organized, smooth, and uniform (i.e., contain minimal defects), similar to those on gold. Since the reactive hydroxyl groups on amorphous SiO_2_ surfaces are randomly distributed, increasing the fraction of monomers bonded to these sites is expected to force the monolayer into an increasingly disordered structure [37]. This study suggests that fully or partially physisorbed monolayers can be decoupled from their underlying surfaces to a degree, allowing for the in-plane lateral reorganization of monomers into a more ordered configuration; chemisorbed monolayer structure, however, is predominantly determined by surface structure. Thus, increasing the fraction of chemisorbed chains may lead to increased coefficients of friction and adhesion, as prior studies have reported a negative correlation between friction and monolayer ordering [16,23,24,25]. However, covalent bonds anchoring chains to the surface are thought to be necessary for durability/robustness [48], so decreasing the fraction of chemisorbed chains could cause the monolayer to degrade over shorter periods of time. Such behavior was observed by Booth et al., who compared the frictional properties of physisorbed *n*-alkanethiols on gold with chemisorbed *n*-alkylsilanes on oxidized silicon, finding that the physisorbed monolayers exhibited a threefold improvement in coefficient of friction at low normal loads, while the chemisorbed monolayers exhibited significantly improved durability and were able to withstand normal loads at least 30 times larger than those that damaged the physisorbed monolayers [17].

Direct control over surface morphology and decoupling of the numerous factors that influence friction and wear in monolayer systems is nearly impossible through a purely experimental approach. Computational methods, including molecular dynamics (MD) simulations, have emerged as an important tool to probe the molecular-level behavior of nanotribological systems, and recent improvements in computational speed and modeling methods have made simulations that closely mimic experimental systems possible. For example, MD simulations have been applied to improve our understanding of alkylsilane monolayers on oxidized surfaces [14,16,23,24,51,52,53,54,55,56,57,58,59,60,61], providing insight into the effects of various monolayer properties on frictional behavior, including monomer structure (e.g., backbone [14,55] and terminal group [13,16]) and surface structure (e.g., roughness and density of surface-bound hydroxyl groups) [53,54,55,57,60]. However, the simulations of alkylsilane monolayers performed to date have examined systems without siloxane cross-linkages. Furthermore, most have considered only fully chemisorbed monolayers; notable exceptions include the work of Chandross et al., who studied the behavior of fully and partially physisorbed monolayers under shear by nanometer-scale tips in order to imitate the process by which atomic force microscopes measure forces between the tip and sample [57,58,59], and our previous work studying monolayer degradation under shear, in which interfacial Si–O bonds (i.e., those connecting chemisorbed monomers to the surface) were severed at random and the mobility of the broken chains examined [61]. While excluding cross-linkages seems like a reasonable assumption, it introduces an approximation compared to the experimental systems and is often cited as the reason for discrepancies observed in monolayer properties between the simulations and experiments. In its role as a stabilizer, cross-polymerization could potentially influence monolayer structure in measurable ways (e.g., packing density, orientational and/or conformational ordering, monomer tilt, monolayer surface roughness, or number of defects). Furthermore, does any observed effect depend on the fraction of monomers covalently bonded to the surface? For example, physisorbed monolayers can be somewhat decoupled from surfaces [37], possibly enabling cross-polymerization to play a more significant role than in their chemisorbed counterparts. These questions remain unanswered and are of particular concern given that previous studies have reported correlations between alkylsilane monolayers’ structural properties and their frictional performance [23,25,53,60].

In an effort to address these fundamental questions regarding alkylsilane monolayer structure and cross-polymerization, we have developed two different simulation procedures to construct fully and partially chemisorbed *n*-alkylsilane (CH_3_(CH_2_)*_n_*_−1_Si(OH)_3_) monolayers featuring cross-linkages on amorphous silica surfaces. The structural properties and frictional performance of these cross-polymerized monolayers have been assessed and compared to those of equivalent monolayers without cross-linkages in order to isolate any effects of (1) siloxane cross-linkages and (2) the fraction of chains covalently bonded to the surface.

## 2. Simulation Methods

### 2.1. Initialization of Cross-Polymerized Monolayers

As discussed above, most previous simulations of alkylsilane monolayers on silica substrates have considered fully chemisorbed monolayers without siloxane cross-linkages [14,16,23,24,51,52,53,54,55,56,60]. Comparisons to equivalent cross-polymerized monolayers (i.e., those with all chains covalently bonded to the surface) would isolate any changes to structural properties and/or frictional performance that are directly correlated with cross-linkages. However, cross-polymerization is more likely to affect structure if fewer monomers are bonded to the surface; thus, monolayers in which not all chains are directly chemisorbed must also be considered. Here, two different approaches have been taken to create fully and partially chemisorbed *n*-alkylsilane monolayers featuring cross-linkages on amorphous silica surfaces. The methods used to initialize these systems were developed with the overall goal of creating monolayers that closely match those prepared experimentally. As such, they are essentially different implementations of the same underlying idea, guided by a common philosophy: cross-polymerized monolayers are assembled in a stepwise manner through random processes, restricted only by steric effects. Both methods yield packing densities that are consistent with experimental *n*-alkylsilane monolayers assembled on silica (~4.0–5.0 chains/nm^2^) [47,52,62,63], as well as cross-linkages that are consistent with the proposed instantaneous structure of an alkylsilane monolayer in a state of dynamic equilibrium (i.e., small linear or cyclic oligomers of crosslinked monomers (≤6), most of which are dimers and trimers) [40].

#### 2.1.1. Chemisorbed Monolayers

**Figure 2:**
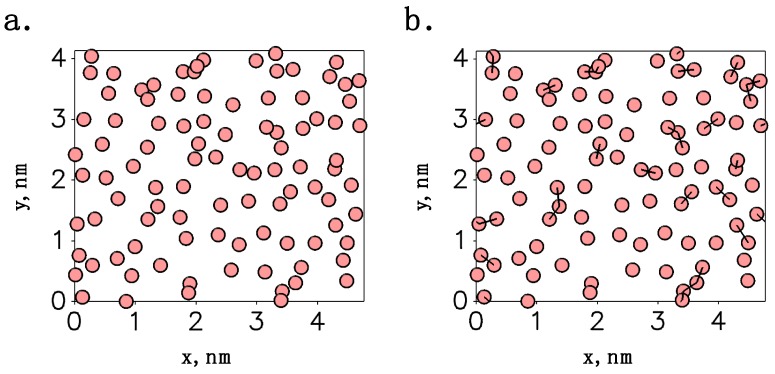
Chemisorbed monolayers with cross-linkages constructed using a two-stage procedure, whereby (**a**) a chemisorbed alkylsilane monolayer without cross-linkages is assembled on an amorphous silica surface with a high density of surface-bound hydroxyl groups (~5.8 OH/nm^2^), and (**b**) cross-linkages are inserted at random between neighboring chains in the monolayer. Spheres represent the silicon atoms in alkylsilane head groups. These images and others in this work were generated using the Visual Molecular Dynamics (VMD) software (version 1.9.3, Theoretical and Computational Biophysics Group (University of Illinois at Urbana-Champaign), Urbana, IL, USA) [64].

To facilitate direct comparison with previous simulations [14,16,23,24,51,52,53,54,55,56,60], cross-polymerized alkylsilane monolayers in which all monomers are covalently bonded to the surface have been created. Fully chemisorbed monolayers featuring cross-linkages have been constructed by the procedure summarized in Figure 2. A previously developed synthesis mimetic simulation (SMS) procedure was initially used to generate amorphous silica with a high density of surface-bound hydroxyl groups and then to attach a chemisorbed alkylsilane monolayer without cross-linkages (see Figure 2a) [60]. The SMS procedure was designed to mimic the postsynthesis processing of silicon wafers with “piranha” solution (H_2_SO_4_/H_2_O_2_), which is done in experiments to encourage the chemisorption of alkylsilanes during monolayer formation [23]. The process results in surfaces with atomic-scale surface roughness (root-mean-squared roughness of ~0.13 nm) and a dense layer of surface-bound hydroxyl group bonding sites for chains (~5.8 OH/nm^2^); root-mean-squared roughness was estimated by the standard deviation of the positions of oxygen atoms that are part of surface-bound hydroxyl groups in the direction normal to the surface plane (i.e., in the *z*-direction). Monolayers with varying densities (3.9–4.9 chains/nm^2^) were then generated by varying the minimum cutoff distance between bonding sites from 2.0 to 2.5 Å and attaching monomers. Full details of the procedure can be found in the original paper [60]. To create the final cross-polymerized monolayers, siloxane (Si–O–Si) linkages were inserted between neighboring monomers (see Figure 2b). Cross-linkages are observed to have a length of ~2.8–3.3 Å [40], so monomers separated by ≤3.3 Å were considered eligible pairs for bonding. Cross-linkages were inserted to connect eligible pairs at random, with the restriction that each monomer can only bond with up to two neighbors. As an example, a system with 96 chains at a density of 4.9 chains/nm^2^ is shown in Figure 2b. As can be seen from the figure, its 28 cross-linkages are scattered throughout the monolayer, creating small clusters of linked chains (i.e., 12 dimers, 6 trimers, and 1 linear oligomer of 5 chains). Note that this extent/organization of cross-linkages is consistent with the proposed instantaneous structure of an alkylsilane monolayer in a state of dynamic equilibrium (i.e., small oligomers of cross-linked monomers (≤6), most of which are dimers and trimers) [40].

#### 2.1.2. Partially Chemisorbed Monolayers

**Figure 3:**
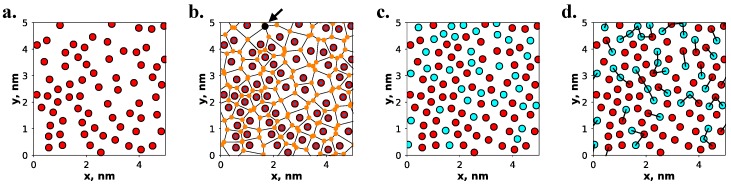
Partially chemisorbed monolayers are constructed using a multistage process whereby (**a**) chemisorbed chains are placed in an arrangement to fill all available surface sites without steric overlaps (using a van der Waals diameter of 0.42 nm per chain [65]), (**b**) Voronoi tessellation is used to determine locations for additional insertion of chains not bound to the surface (the arrow designates the site used for the first chain insertion), and this procedure is repeated until (**c**) no additional available locations exist and the monolayer is considered complete. Finally, (**d**) inserted chains that are not surface bound have bonds drawn to neighboring chains to create a cross-linked network. Red spheres represent the silicon atoms in chemisorbed chains and cyan spheres represent those in chains attached only via cross-linkages.

As previously mentioned, cross-polymerization may play a more significant role in the behavior of alkylsilane monolayers if fewer monomer chains are bonded to the surface. To examine these effects, an additional set of monolayer systems has been studied in which only a fraction of chains is chemisorbed to the surface, while the remainder are bonded only via cross-linkages to other chains. Recall that these partially chemisorbed monolayers are more common in real systems than fully chemisorbed or physisorbed monolayers as a result of the island growth mechanism by which they form [30,31]. The procedure used to construct these systems features two stages, as shown in Figure 3, and is controlled purely by sterics.

As with the fully chemisorbed monolayers, amorphous silica substrates were used to construct the partially chemisorbed monolayer systems. Using the mBuild toolkit [66], an analytical method was used to generate amorphous surfaces through carving from a bulk silica slab and adjusting the hydroxyl density to 5 OH/nm^2^, to match expectations from experiment [37,39], by bridging neighboring surface oxygen atoms [67,68]. We note that the SMS procedure was not used for the partially chemisorbed monolayer systems since maximizing the density of surface-bound hydroxyl groups is not necessary for these systems, and so a more traditional, less computationally intensive approach to surface generation was taken. This method allows greater control over the initialization of the film structure needed to construct the partially chemisorbed monolayers. Substrates generated using this simpler approach featured a surface roughness of ~0.11 nm, thus closely approximating the structure of the SMS-generated surfaces. The initial stage of monolayer creation, as shown in Figure 3a, involved the placement of the chains that were directly bonded to the surface. Thus, only the discrete locations of surface hydroxyls on the substrate acted as available sites for attaching chains. In an iterative fashion, an available site was chosen at random, a chain was then placed at this location, and the list of available sites was updated to ensure that future chains would not overlap with existing chains. When no available sites remained, the chemisorbed portion of the monolayer was considered complete; however, if a desired number of chemisorbed chains was explicitly specified, then additional chains were added at locations that featured the furthest distance from existing chains. The second stage of monolayer construction, shown in Figure 3b,c, considered the placement of chains that were attached to the surface only through cross-linkages to other chains. To determine the locations for these chains, an iterative procedure was used whereby a 2D Voronoi tessellation was performed on the set of points representing the locations of existing monolayer chains. A new chain was placed at the site of the Voronoi vertex featuring the furthest distance from any existing monolayer chain. This process was repeated until either no locations exist, whereby overlap with existing chains occurred, or a desired total number of monolayer chains was reached. Each newly inserted chain was then attached via a cross-linkage to its nearest neighbor, which may be directly bonded to the surface or via a cross-linkage to another chain. The code used to construct these monolayers is available online (see Supplementary Materials). Constructing alkylsilane monolayers via this procedure yielded an average density of 3.9 ± 0.1 chains/nm^2^ which was in close agreement with monolayer densities estimated from experiment (4.0–5.0 chains/nm^2^) [47,52,62,63]. Additionally, this procedure resulted in monolayers featuring roughly 65% chemisorbed chains, with the remaining 35% of chains attached via cross-linkages only. It is of importance to note that the monolayer density of the chemisorbed chains (2.5 chains/nm^2^) agreed well with the density observed experimentally for alkanol molecules (which cannot form cross-linkages but feature a comparable van der Waals (VDW) diameter) attached to amorphous silica (2.65 chains/nm^2^) [69].

### 2.2. Molecular Dynamics Simulations

Molecular dynamics simulations of fully and partially chemisorbed alkylsilane monolayers have been performed under equilibrium and nonequilibrium conditions. Postequilibration trajectory lengths ranged from 1–3 ns for equilibrium simulations and 5–10 ns for nonequilibrium simulations. These trajectory lengths were found to be sufficient in order for the simulations to converge to a steady state and to yield data with reasonably low uncertainty. Simulations were conducted using the optimized potentials for liquid simulations all-atom (OPLS-AA) force field [70]. The OPLS-AA parameters used for this work were taken from Lorenz et al. [54] for silica and Jorgensen et al. [70] for alkanes (see Supplementary Materials for details), in accordance with prior simulation studies of alkylsilane monolayers on silica [14,16,23,24,60,61]. All simulations were performed in the canonical, or *NVT*, ensemble (i.e., constant number of atoms, volume, and temperature) at a temperature of 298.15 K, with periodic boundary conditions in the surface plane (i.e., the *xy*-plane) in order to mimic the behavior of an infinite surface; simulations did not interact across the *z*-boundary. Shearing speeds of 10 m/s were used. While this speed is several orders of magnitude larger than those normally used in experiments (e.g., atomic force microscopy and tribometry, where shear rates are typically on the order of micrometers per second [17]), several studies report that shearing velocities of this magnitude and higher do occur between surfaces in nanotribological systems, including MEMSs/NEMSs [6,14,52,53,54,55,71]. Furthermore, prior studies have shown that frictional forces do not significantly depend on sliding velocity at moderate loads [14,52,53,54,55]. For these simulations, thermostatting was not performed in the direction of shear to allow for the possibility of viscous heating, although appreciable shear-induced heating has not been observed at the shear rates considered here [61]. Additional details regarding the simulation procedures are provided in the Supplementary Materials [72,73,74,75,76,77,78,79,80,81].

In order to quantify the structural properties of all monolayer systems considered in this work, the nematic order parameter (*S*_2_), average tilt angle (*θ*), and gauche defect fraction were calculated under equilibrium and nonequilibrium conditions. The nematic order parameter was used to quantify global orientational ordering in each layer. A value of *S*_2_ = 1 indicates perfect orientational ordering within a monolayer, and values of *S*_2_ less than unity represent proportionately less ordering [82,83]. Here, values of *S*_2_ below ~0.8 indicated a distinct loss of orientational ordering, as determined via visual inspection. Note that since these molecules are attached to a surface, the transition from well-ordered to disordered occurs at a higher value of *S*_2_ than is typically seen for bulk nematic systems [83]. Average tilt angle is defined such that monolayers in perfect alignment with a vector normal to the silica surface yield a tilt angle of 0°. Monolayers’ gauche defects were assessed via the gauche defect fraction; in this calculation, a dihedral angle (i.e., the twist of a C–C–C–C quadruplet in the monomer backbone) was considered to be in the trans state if it was between 90° and 270°, while angles outside this range were considered to be gauche defects [84]. A detailed description of the calculation of each of these metrics is provided in the Supplementary Materials [82,83,84,85]. To quantify frictional performance, the coefficient of friction (*μ*) and adhesion/force intercept (*F*_0_) were determined for all monolayers undergoing shear. Specifically, *μ* and *F*_0_ were calculated according to a modified form of Amontons’s Law of Friction [20,86,87]:

(1) $$F_{f}=\mu F_{n}+F_{0}$$

where *F_f_* represents the friction force, *F_n_* represents the normal force, and *F*_0_ represents the extrapolated friction force at zero load, or adhesion/force intercept. Simulations were conducted at several different normal loads, and *μ* and *F*_0_ were approximated by the slope and *y*-intercept of the line generated by plotting friction force as a function of normal force, respectively.

## 3. Results and Discussion

**Figure 4:**
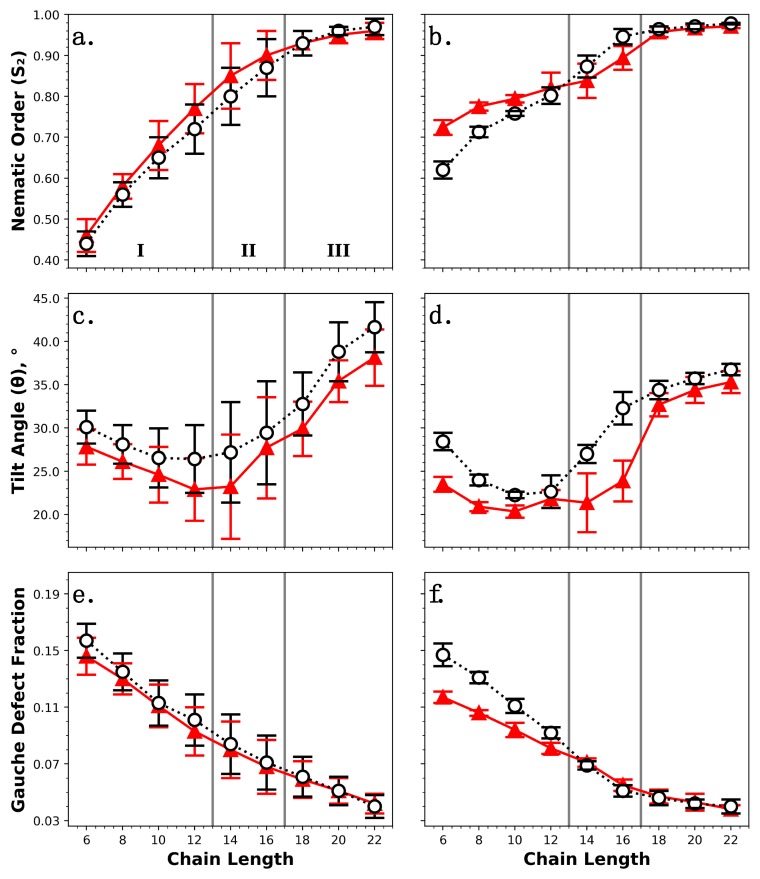
Structural properties of monolayers with or without cross-linkages (black circles and red triangles, respectively) as a function of chain length; the three chain-length-dependent regions described in the text are indicated by I, II, and III. Fully chemisorbed monolayers with or without cross-linkages are compared in (**a**,**c**,**e**) (error bars represent the standard deviation for three unique monolayer systems), and partially chemisorbed monolayers featuring cross-linkages are compared to equivalent fully chemisorbed monolayers without them in (**b**,**d**,**f**) (error bars represent the standard deviation for five unique monolayer systems). Structure was assessed via nematic order parameter (*S*_2_) (**a**,**b**), average tilt angle (*θ*) (**c**,**d**), and gauche defect fraction (**e**,**f**). Lines connecting data points are provided only to guide the eye. This plot and the others included in this work were generated using the python plotting library matplotlib [88].

To ascertain the impact of cross-polymerization and surface attachment on the structural properties of alkylsilane monolayers, equilibrium simulations have been performed for all systems. The nematic order parameter (*S*_2_), average tilt angle (*θ*), and gauche defect fraction have been determined as a function of alkylsilane backbone length (6–22 carbon atoms) and compared for monolayers with or without cross-linkages. The results are summarized in Figure 4, with Figure 4a,c,e comparing *S*_2_, *θ*, and the gauche defect fraction, respectively, for fully chemisorbed monolayers with cross-linkages (see Figure 2b) and identical chemisorbed monolayers without cross-linkages (see Figure 2a). The same comparisons are made in Figure 4b,d,f for partially chemisorbed monolayers with cross-linkages (see Figure 3d) and systems with all chains chemisorbed and no cross-linkages; note that this comparison also features the fraction of chains chemisorbed to the surface as a second variable. The four systems studied are summarized in Table 1. The transition from a disordered, liquid-like state to one that was well-ordered/solid-like occurred when *S*_2_ increased to ~0.8, which was observed for all four monolayer systems as chain length increased from 14 to 16 carbon atoms (see Figure 4a,b). Thus, as highlighted in Figure 4, three chain-length-dependent regions appeared to exist: (I) a liquid-like region (chain length < 14), indicated by lower nematic order and more gauche defects per chain; (II) a transition region (14 ≤ chain length ≤ 16), where monolayers transitioned between liquid-like and solid-like states and may have featured local regions of order (indicated by larger error bars for global monolayer properties); and (III) a solid-like region (chain length > 16), indicated by high values of nematic order and a low number of gauche defects per chain.

As shown in Figure 4a,b, *S*_2_ increased with monomer length for all systems with or without cross-linkages. A positive correlation between monolayer ordering and monomer length has been observed previously in experiments [23,24,25] and simulations [16,23,24,60]. This trend can be explained by an increase in stabilizing VDW forces between chains as the number of backbone carbon atoms was increased. Our initial expectation was that the addition of cross-linkages would increase orientational ordering, as neighboring monomers connected by siloxane linkages would be forced into closer proximity; furthermore, experimental results suggest that reducing the fraction of monomers that are chemisorbed to the surface also increases global ordering, as nonchemisorbed monomers are not coupled to the locations of surface-bound hydroxyl groups [37]. However, the results shown in Figure 4a,b suggest that both cross-linkages and the ratio of chemisorbed to physisorbed chains play a more complex role in monolayer ordering, which appears to vary as a function of monomer length. Figure 4b shows that the partially chemisorbed monolayers constructed from short chains (region I) had reduced ordering compared with the equivalent chemisorbed monolayers without cross-linkages. Although the same effect is observed in Figure 4a, the differences are not statistically significant, and thus, it is likely related specifically to the fraction of chains chemisorbed to the surface rather than cross-linkages. This observation is surprising, as reducing the number of chemisorbed chains is expected to result in a film that features greater in-plane fluidity (i.e., chains should be better able to rearrange themselves in the surface plane), thus allowing the chains to adopt a more uniform configuration. However, it is also known that the VDW forces that provide cohesiveness to monolayer films are weaker for shorter chains, and it appears that the lack of strong cohesive VDW forces takes precedence over the increase in surface plane mobility when determining monolayer ordering for these systems. In Figure 4b, the curves for partially chemisorbed monolayers and their fully chemisorbed counterparts have different slopes in regions I and II, and as a result, a transition occurs at a chain length of 14, whereby the partially chemisorbed monolayers exhibit higher ordering than their chemisorbed analogues. This phenomenon is not observed in Figure 4a, which once again suggests that it was related to the increase in surface plane mobility when fewer monomers were chemisorbed to the surface; at chain lengths of 14–16 (region II), the cohesive VDW forces were then strong enough that increased mobility did in fact lead to a more ordered monolayer. Figure 4a suggests that fully chemisorbed monolayers with cross-linkages were slightly less ordered than those without them for all chain lengths below 18 (regions I and II), which may have been due to the fact that Si–O–Si cross-linkages were too short to allow the aliphatic tails to be parallel without overlapping; as a result, chains connected by cross-linkages would tilt or bend/twist away from each other, causing their tails to be splayed apart and leading to a slightly more disordered monolayer. As chain length increased to 18 and above (region III), all monolayer systems described in Figure 4a,b existed in a highly ordered, solid-like state (*S*_2_ > 0.95), where neither cross-linkages nor the fraction of chemisorbed chains appeared to have a significant influence on nematic ordering.

Figure 4c,d indicate that there is a correlation between alkylsilane backbone length and average tilt angle, where *θ* is minimized for systems with intermediate monomer lengths just below the transition from a disordered to well-ordered state (10–14). This observation can be explained by referring back to the results for *S*_2_ (Figure 4a,b). Monolayers with the shortest chain length of 6 backbone carbon atoms existed in a highly disordered state, where chains were allowed to adopt various tilted conformations with no preferred orientation. As chain length increased to 14, the cohesive VDW forces became stronger and caused the chains to stand more upright. When chain length exceeded 14, the chains once again adopted tilted conformations, except now with a preferred orientation (i.e., chains tilted in the same direction) in order to maximize film cohesivity. The inclusion of cross-linkages appeared to increase the average tilt angle at all chain lengths by ~1°–5° in chemisorbed monolayers (Figure 4c) and ~1°–10° in partially chemisorbed monolayers (Figure 4d). Since this effect is more pronounced in Figure 4d, it is likely related to both cross-linkages and the fraction of chains chemisorbed to the surface. Adding cross-linkages may have led to an increase in overall tilt because cross-linked chains must tilt away from each other to prevent their tails from overlapping, as previously mentioned. Any further increase in *θ* when fewer chains were bonded directly to the surface was likely related to the mobility of the nonchemisorbed chains in the surface plane.

Figure 4e,f suggest that all of the monolayer systems with or without cross-linkages featured most C–C–C–C dihedrals in the trans state, with only a few gauche defects. This observation is consistent with experimental results for densely packed alkylsilane monolayers on silica (≤4% gauche defects) [35,69]. The number of defects appeared to decrease with increasing chain length, a trend that has been observed previously in experiments [69] and simulations [51,52]. This effect can be explained by once again referring to the results for *S*_2_ (Figure 4a,b). Monolayers with short chain lengths existed in a disordered state (region I), allowing both monomer tilting and gauche distortions (i.e., twisting about the C–C bond axis) to occur more easily. As chain length was increased and the monolayer became more well-ordered, fewer defects were able to form. The presence of cross-linkages did not appear to have a meaningful impact on the gauche defect fraction for systems in which all chains were chemisorbed to the surface (Figure 4e). Recall that adding cross-linkages to chemisorbed monolayers led to a small increase in average tilt angle at all chain lengths (Figure 4c). These results combined may indicate that cross-linked chains prefer to tilt away from each other rather than contort via gauche deformations when avoiding overlaps between their aliphatic tails. Partially chemisorbed monolayers constructed from chains with less than 14 backbone carbon atoms (region I) had a larger number of gauche defects than equivalent chemisorbed monolayers without cross-linkages (Figure 4f); this result is somewhat expected, given that the partially chemisorbed monolayer systems in region I also exhibited reduced nematic ordering (Figure 4b). The effect is most likely related to the fraction of chains bonded to the surface rather than cross-linkages (as it is not observed in Figure 4e), and more specifically, to the mobility of nonchemisorbed chains in the surface plane. Chains that are not directly coupled to bonding sites on the surface can more easily tilt and/or contort via gauche deformations, as discussed previously.

**Figure 5:**
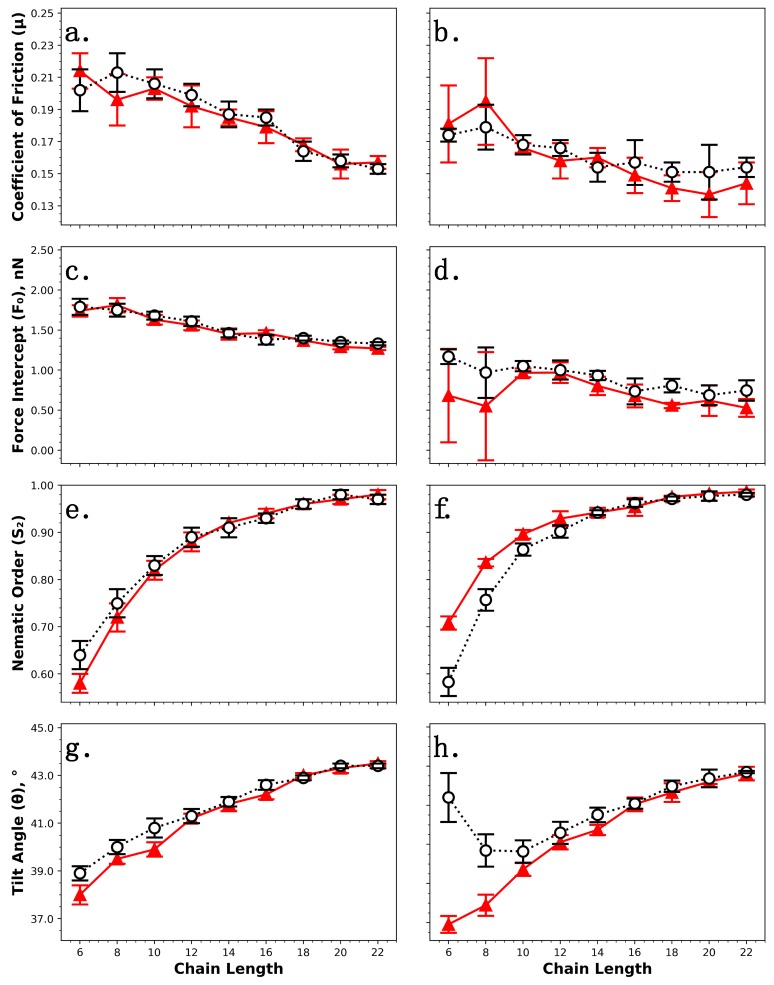
Tribological and structural properties of monolayers with or without cross-linkages (black circles and red triangles, respectively) under shear as a function of chain length. Fully chemisorbed monolayers with or without cross-linkages are compared in (**a**,**c**,**e**,**f**) (error bars represent the standard deviation for three unique monolayer systems), and partially chemisorbed monolayers featuring cross-linkages are compared to equivalent fully chemisorbed monolayers without them in (**b**,**d**,**f**,**h**) (error bars represent the standard deviation for five unique monolayer systems). Tribology is assessed via coefficient of friction (*μ*) (**a**,**b**) and force intercept (*F*_0_) (**c**,**d**), and structure is assessed via nematic order parameter (*S*_2_) (**e**,**f**) and average tilt angle (*θ*) (**g**,**h**). Lines connecting data points are provided only to guide the eye.

To assess the tribological impact of cross-linkages within alkylsilane monolayers, nonequilibrium molecular dynamics simulations have been performed to examine the frictional performance and structural properties of monolayers under shear. As discussed previously, experimental studies have attributed alkylsilane monolayers’ tribological performance (i.e., robustness under harsh conditions and ability to protect underlying surfaces from damage) to their capacity to form strong covalent bonds with both the surface and each other [17,19]. The coefficient of friction (*μ*) and force intercept (*F*_0_) have been calculated via Equation (1) to quantify frictional performance, and the nematic order parameter (*S*_2_) and tilt angle (*θ*) have been calculated to assess structural properties; these metrics are again determined as a function of alkylsilane backbone length (6–22) and compared for monolayers with or without cross-linkages. The results are summarized in Figure 5. Chemisorbed monolayers with cross-linkages (see Figure 2b) are compared to identical chemisorbed monolayers without them (see Figure 2a) in Figure 5a,c,e,g, while partially chemisorbed monolayers with cross-linkages (see Figure 3d) are compared to fully chemisorbed systems without cross-linkages in Figure 5b,d,f,h; once again, note that this comparison also features the fraction of chains chemisorbed to the surface as a second variable.

These results presented for the coefficient of friction (Figure 5a,b) and force intercept (Figure 5c,d) suggest that neither cross-linkages nor the fraction of chemisorbed/physisorbed chains play a noticeable role in frictional performance, as both the chemisorbed (Figure 5a,c) and partially chemisorbed (Figure 5b,d) monolayer systems yielded similar results compared with their analogues without cross-linkages. Partially chemisorbed monolayers did appear to yield slightly higher force intercepts than their chemisorbed counterparts without cross-linkages, but this result was not statistically significant at most chain lengths (Figure 5d). The results presented here suggest that excluding cross-linkages, as was done in prior simulations of alkylsilane monolayers, is a reasonable assumption, at least at short time scales; however, we note that both cross-linkages and covalent surface attachment are expected to play a more important role in frictional performance over longer periods of time than can be considered by simulation, especially under conditions that facilitate degradation and wear.

Figure 5a,b show that *μ* decreased with monomer chain length for all systems with or without cross-linkages, while Figure 5c,d show that *F*_0_ also generally decreased, with the exception of partially chemisorbed monolayers with six to eight backbone carbon atoms (Figure 5d). Prior experiments [23,24,25] and simulations [16,23,24,60] have reported that adhesion and friction decrease with increasing chain length, a trend which was attributed to increased cohesivity (and thus a higher degree of ordering) in monolayers constructed from longer chains. This explanation is supported by Figure 5e,f, which show that *S*_2_ increased with chain length for all systems with and without cross-linkages. Recall that a positive correlation between monomer length and *S*_2_ was also observed for monolayer systems at equilibrium (see Figure 4a,b); however, the three chain-length-dependent regions which were present at equilibrium were not observed for the same monolayer systems under shear. *S*_2_ increased under shearing conditions (as compared with equilibrium) for all of the fully chemisorbed monolayers, both with and without cross-linkages, and all partially chemisorbed monolayers except for the C_6_ system; this observation is attributable to forced shear alignment of the chains.

Figure 5f indicates that partially chemisorbed monolayers constructed from chain lengths ≤ 10 had reduced ordering compared with equivalent chemisorbed systems without cross-linkages; this effect is not observed in Figure 5e, which suggests it is related specifically to the fraction of monomers chemisorbed to the surface and not cross-linkages. A more striking structural difference is visible in Figure 5h; partially chemisorbed monolayers with chain lengths ≤ 10 yielded tilt angles that were significantly higher than those of their fully chemisorbed counterparts, where the effect was most pronounced for the shortest backbone chain length of six carbon atoms. Again, this trend is not observed in Figure 5g, so it is likely related to the fraction of chemisorbed chains rather than cross-linkages. For all of the fully chemisorbed monolayer systems with and without cross-linkages shown in Figure 5g,h, the average tilt angle was higher under shearing conditions (as compared with equilibrium) and also increased with chain length; this trend was also observed for all of the partially chemisorbed systems with chain lengths > 10 (Figure 5h). These results seem to suggest a lack of shear alignment for the partially chemisorbed monolayer systems with short chain lengths (6–10), which would also explain the reduced nematic ordering for these systems (Figure 5f).

**Figure 6:**
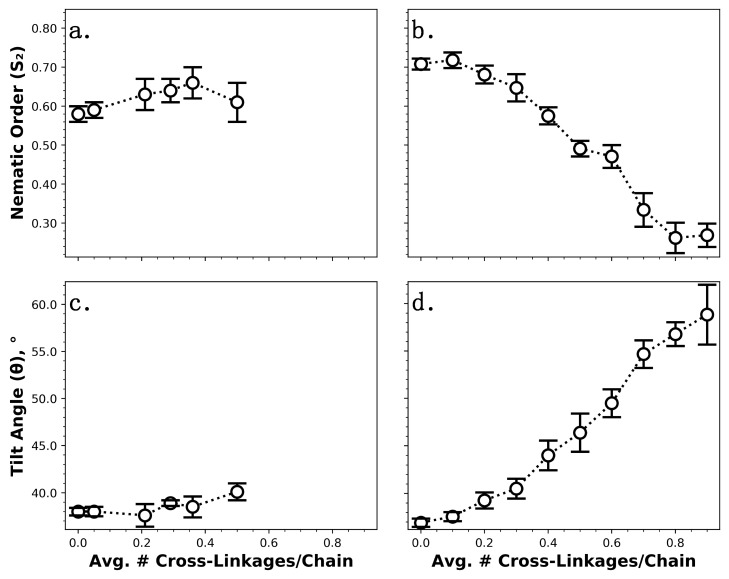
Structural properties of C_6_ monolayers under shear as a function of the average number of cross-linkages per chain. Structure was assessed via nematic order parameter (*S*_2_) (**a**,**b**) and average tilt angle (*θ*) (**c**,**d**). Fully chemisorbed monolayers with cross-linkages are compared in (**a**,**c**) and partially chemisorbed monolayers featuring cross-linkages are compared in (**b**,**d**). Lines connecting data points are provided only to guide the eye.

To further investigate the apparent discrepancy in structure between partially chemisorbed monolayers and their fully chemisorbed counterparts without cross-linkages at short chain lengths (≤10), fully and partially chemisorbed C_6_ monolayer systems with varying numbers of cross-linkages have also been studied (Figure 6). We note that it is not possible to create fully chemisorbed monolayers with more than ~0.5 cross-linkages per chain due to the requirements that (1) chemisorbed chains must be coupled to hydroxyl groups on the silica surface that are separated by ≥2.0 Å to avoid steric hindrance [60], and (2) cross-linked chains must be separated by a maximum of 3.3 Å because siloxane (Si–O–Si) linkages are observed to have a length of ~2.8–3.3 Å [40]. As shown in Figure 6a,c, the number of cross-linkages did not appear to have a significant impact on *S*_2_ or *θ* for C_6_ monolayers in which all chains were chemisorbed to the surface; however, some structural differences were observed for the partially chemisorbed C_6_ monolayers, as shown in Figure 6b,d. As the number of cross-linkages per chain increased (and thus the fraction of chemisorbed chains decreased), *S*_2_ decreased dramatically and *θ* increased dramatically. These results combined indicate a lack of shear alignment for partially chemisorbed C_6_ monolayers, which becomes more pronounced as the fraction of chains chemisorbed to the surface decreases. The values of *S*_2_ and *θ* for partially chemisorbed C_6_ monolayers reached those of analogous fully chemisorbed monolayers when the fraction of chemisorbed chains exceeded ~0.9 (i.e., the average number of cross-linkages per chain dropped below ~0.1) (see Figure 5b,d); at this point, the monolayers are expected to be in a forced orientation due to shear alignment.

**Figure 7:**
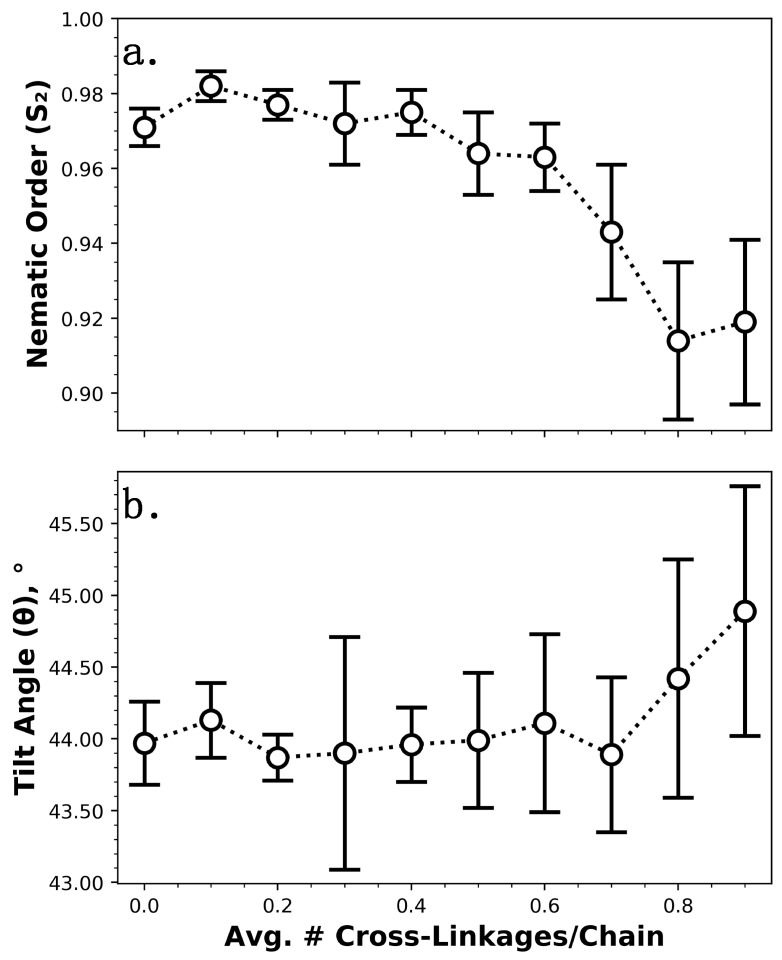
Structural properties of partially chemisorbed C_18_ monolayers under shear as a function of the average number of cross-linkages per chain. Structure was assessed via (**a**) nematic order parameter (*S*_2_) and (**b**) average tilt angle (*θ*). Error bars represent the standard deviation for five trials (each with a unique monolayer configuration) and lines are provided only to guide the eye.

We note that about two-thirds of the chains in the partially chemisorbed monolayers described previously in Figure 5 were bonded to the surface, so given the results in Figure 6, it seems plausible that decreasing the fraction of chemisorbed chains has little impact on structural properties up to a certain point for monolayers constructed from longer chains (>10 backbone carbon atoms). To examine this idea, partially chemisorbed C_18_ monolayers with varying numbers of cross-linkages have been studied (Figure 7); note that C_18_ monolayers are also more commonly used in experimental systems and applications, as they are more stable and thus better able to reduce stiction and protect surfaces from wear [24,89]. As can be seen from Figure 7, the values of *S*_2_ and *θ* for these systems reached those of analogous fully chemisorbed monolayers when the fraction of chemisorbed chains exceeded ~0.4 (i.e., the average number of cross-linkages per chain dropped below ~0.6, see Figure 5b,d), a value which was significantly lower than that for the C_6_ systems. Furthermore, the overall changes in structure for the C_18_ systems were minimal compared with those in the C_6_ systems (an overall spread of ~0.07 in *S*_2_ compared with ~0.46, and ~1° in *θ* compared with ~22°). These results indicate that reducing the fraction of chains chemisorbed to the surface has a minimal effect on monolayer structure under shear until a certain threshold is reached, after which the chains become increasingly disordered and exhibit increasing average tilt due to a lack of shear alignment; this threshold appears to decrease with increasing chain length, and is below about two-thirds for chain lengths of >10 backbone carbon atoms. For C_18_ systems, which are most industrially relevant [24,89], neither cross-linkages nor the fraction of chemisorbed/physisorbed chains appear to play any significant role in monolayer structure under shearing conditions.

## 4. Conclusions

In this work, fully and partially chemisorbed *n*-alkylsilane (CH_3_(CH_2_)*_n_*_−1_Si(OH)_3_) monolayers featuring cross-linkages on amorphous silica surfaces have been studied. The structural properties and frictional performance of these cross-polymerized monolayers have been assessed and compared to those of equivalent monolayers without cross-linkages in order to isolate the impact of (1) siloxane cross-linkages and (2) the fraction of chains covalently bonded to the surface.

Equilibrium simulations used to ascertain the effects of cross-polymerization on structural properties showed that both cross-linkages and the ratio of chemisorbed to physisorbed chains affect monolayer structure in small but measurable ways that vary based on chain length. Three chain-length-dependent regions were observed: (I) a liquid-like region (chain length < 14), (II) a transition region (14 ≤ chain length ≤ 16), and (III) a solid-like region (chain length > 16). Fully chemisorbed monolayers with cross-linkages exhibited slightly reduced nematic ordering compared with those without them in regions I and II, likely due to the cross-linked chains tilting or bending/twisting away from each other to prevent overlaps and causing their tails to be splayed apart, leading to a slightly more disordered monolayer. Chemisorbed monolayers with cross-linkages also yielded slightly higher average tilt angles than their counterparts without them, but cross-linkages did not play a meaningful role in the formation of gauche defects. These observations combined suggest that cross-linked chains prefer to tilt away from each other rather than contort via gauche deformations when avoiding overlaps. Partially chemisorbed monolayers in region I exhibited reduced nematic ordering compared with equivalent chemisorbed monolayers without cross-linkages. Albeit surprising, as reducing the number of chemisorbed chains is expected to result in greater in-plane fluidity, the VDW forces that provide monolayer cohesiveness are weaker for shorter chains and appear to take precedence over the increase in surface plane mobility for these systems. The partially chemisorbed monolayers did exhibit higher ordering than their chemisorbed analogues in region II, which suggests that the VDW forces were strong enough for increased mobility to lead to a more ordered monolayer for these systems. Partially chemisorbed monolayers in regions I and II yielded higher tilt angles than their chemisorbed counterparts, and in region I, they also had more gauche defects. These results can likely be explained by the increased mobility of nonchemisorbed chains in the surface plane; chains that are not directly coupled to bonding sites on the surface can more easily tilt into nonupright conformations and/or contort via gauche defects. In region III, all fully and partially chemisorbed monolayer systems with and without cross-linkages existed in a highly ordered state, where neither cross-linkages nor the fraction of chemisorbed chains appeared to have any significant influence on structural properties.

In the nonequilibrium simulations performed, neither cross-linkages nor the fraction of chemisorbed/physisorbed chains were found to play a noticeable role in frictional performance, as both the chemisorbed and partially chemisorbed monolayers yielded similar results for friction and adhesion compared to their analogues without cross-linkages. The results presented here therefore suggest that excluding cross-linkages, as was done in prior simulation studies, is a reasonable assumption at short time scales (i.e., before degradation and wear must be considered). Under shearing conditions, partially chemisorbed monolayers constructed from short chains (≤ 10 backbone carbon atoms) exhibited reduced nematic ordering and significantly increased chain tilt compared with equivalent chemisorbed systems without cross-linkages. This observation suggests a lack of shear alignment for these systems, which was further investigated by studying partially chemisorbed C_6_ and C_18_ monolayers with varying numbers of cross-linkages (and thus varying numbers of chemisorbed/physisorbed chains). These results indicated that decreasing the fraction of chemisorbed chains has little impact on structural properties up until a certain point that depends on chain length. The overall changes in structure for the C_18_ system were observed to be minimal over the entire range of average cross-linkages per chain considered, which indicates that neither cross-linkages nor the fraction of chemisorbed/physisorbed chains play a significant role in the structure of monolayers constructed from long chains under shear.

## Figures and Tables

**Table 1:** Summary of the key structural properties of the fully and partially chemisorbed monolayer systems studied.

	Cross-Linkages	Average Number Cross-Linkages Per Chain	Fraction of Chemisorbed Chains
**Fully Chemisorbed**	Yes	0.334 ± 0.024	1.000
	No	0.000	1.000
**Partially Chemisorbed**	Yes	0.368 ± 0.015	0.632 ± 0.015
	No	0.000	1.000

## Supplementary Materials

Tabulated force field parameters (Tables S1 and S2), additional details of the simulation methods, and descriptions of the calculations performed to quantify the structural properties of monolayers (Equations (S1)–(S3)) are available online at https://www.mdpi.com/2079-4991/9/4/639/s1. The code used to construct partially chemisorbed monolayers is available online at https://github.com/summeraz/crosslinked_monolayer.
