# Supplementary material for: Investigation of the Impact of Cross-Polymerization on the Structural and Frictional Properties of Alkylsilane Monolayers Using Molecular Simulation
Source: Nanomaterials (Basel). 2019 Apr 19;9(4):639. doi: 10.3390/nano9040639 (PMC6523262; doi:10.3390/nano9040639)
Supplement: Supplementary file 1 [file nanomaterials-09-00639-s001.pdf]

nanomaterials

Article

Supplemental Material for "Investigation of the Impact of Cross-Polymerization on the Structural and Frictional Properties of Alkylsilane Monolayers"

Jana E. Black 1, Andrew Z. Summers 2, Christopher R. Iacovella 3, Peter T. Cummings 4, and Clare McCabe 5,*

Jana E. Black [1], Andrew Z. Summers [1], Christopher R. Iacovella [1], Peter T. Cummings [1] and Clare McCabe [2,*]

[1] Department of Chemical and Biomolecular Engineering, Vanderbilt University, Nashville, TN 37235, USA; jana.black@vanderbilt.edu (J.E.B.); andrew.z.summers@vanderbilt.edu (A.Z.S.); christopher.r.iacovella@vanderbilt.edu (C.R.I.); peter.cummings@vanderbilt.edu (P.T.C.)

[2] Department of Chemical and Biomolecular Engineering and Department of Chemistry, Vanderbilt University, Nashville, TN 37235, USA

* Correspondence: c.mccabe@vanderbilt.edu; Tel.: +1-615-322-6853

Received: 15 March 2019; Accepted: 12 April 2019; Published: 18 April 2019

1. Molecular Dynamics Simulations

Molecular dynamics simulations of fully and partially chemisorbed alkylsilane monolayers featuring cross-linkages have been performed under equilibrium and nonequilibrium conditions. Simulations were conducted using the optimized potentials for liquid simulations all-atom (OPLS-AA) force field [68]. Parameters used for this work were taken from Lorenz et al. [52] for silica and Jorgensen et al. [68] for alkanes. Some minor adjustments were made to these parameters, as detailed in Tables S1 and S2.

Table S1. Bonded parameters.

**Bond Type**	**K_r (kcal mol^{-1} Å^{-2})**		**r_0 (Å)**	
Si–O [52]	300.0		1.650	
Si–C [52]	200.0		1.850	
O–H [52]	553.0		0.945	
C–C [68]	268.0		1.529	
C–H [68]	340.0		1.090	
**Angle Type**	**K_q (kcal mol^{-1} rad^{-2})**		**q_0 (°)**	
Si–O–Si [52]	20.0000		145.000	
Si–O–H [52]	23.7764		122.888	
O–Si–O [52]	60.0000		110.000	
O–Si–C [52]	60.0000		110.000	
Si–C–C [52]	30.4700		120.000	
Si–C–H [68]*	37.5000		110.700	
C–C–C [68]	58.3500		112.700	
C–C–H [68]	37.5000		110.700	
H–C–H [68]	33.0000		107.800	
**Dihedral Type**	**V_1 (kcal mol^{-1})**	**V_2 (kcal mol^{-1})**	**V_3 (kcal mol^{-1})**	**V_4 (kcal mol^{-1})**
C–C–C–C [68]	1.740	-0.157	0.279	0.000
C–C–C–H [68]	0.000	0.000	0.366	0.000
H–C–C–H [68]	0.000	0.000	0.318	0.000

*OPLS-AA parameters not available. K_q and q_0 for C–C–H in alkanes [68] used as an approximation.

Table S2. Nonbonded parameters.

Atom Type	e (kcal mol^{-1})	s (Å)	*q* (*e*)
**Si** (bulk SiO_2) [52]	0.1000	4.0000	+0.860
**O** (bulk SiO_2) [52]	0.1700	3.0000	-0.430
**O** (alkylsilane bonding site on SiO_2 surface) [52]	0.1700	3.0000	-0.215
**H** (SiO**H** on SiO_2 surface) [52]†	0.0000	0.0000	0.000
**Si** (alkylsilane) [52]	0.1000	4.0000	+0.745
**O** (alkylsilane) [52]	0.1700	3.1200	-0.683
**H** (SiO**H**, alkylsilane) [52]	0.0000	0.0000	+0.418
**C** (R**C**H_2R) [68]	0.0660	3.5000	-0.120
**C** (R**C**H_3) [68]	0.0660	3.5000	-0.180
**H** (C**H**$_n$) [68]	0.0300	2.5000	+0.060

†Atomic partial charge (*q*) modified to make systems neutral.

Simulations of chemisorbed alkylsilane monolayers were performed with the LAMMPS simulation engine [70] using a time step of 0.5 fs. Temperature was controlled via the Nosé–Hoover thermostat [71,72] with a temperature damping parameter of 50 ps. The equations of motion were integrated using the multiple time step algorithm rRESPA with time steps of 0.3 fs for bonds, 0.6 fs for angles (valence and dihedral), and 1.2 fs for Lennard-Jones and electrostatic interactions, which were computed using the particle–particle, particle-mesh (PPPM) algorithm for slabs (i.e.,

electrostatic interactions were not calculated across the nonperiodic boundary). Lennard-Jones interactions were computed using a cutoff radius of 10 Å, in accordance with prior simulation studies [14,16,23,24,58,59]. The positions of atoms in the silica surfaces were not integrated through time, so the relative positions of atoms within a substrate remained constant (i.e., the silica substrates behaved as rigid bodies). For the nonequilibrium simulations, monolayer-coated surfaces were first mirrored over the surface plane (i.e., the *xy*-plane) to create two opposing monolayers, which were then compressed at a rate of 1 m/s until coming into contact. Four simulation snapshots were taken from each compression trajectory and used as starting configurations for independent shearing runs at fixed separation distances. Constant velocities of +5 and -5 m/s were then applied to the upper and lower silica surfaces, respectively, in the *x*-direction. The friction force (i.e., sum of all forces in the *x*-direction) and normal load (i.e., sum of all forces in the *z*-direction) on each monolayer-coated surface were determined periodically over time.

Partially chemisorbed alkylsilane monolayers were parameterized using the Foyer atom-typing package [73], and simulations were performed using the Gromacs molecular dynamics engine (v5.1.0) [74]. Hydrogen bonds were held fixed using the LINCS algorithm [75], removing high frequency atomic motions, allowing for a time step of 2.0 fs to be utilized. Temperature was controlled via the Nosé–Hoover thermostat [71,72] with a time constant of 1.0 ps. Equations of motion were calculated using a leap-frog integrator [76], and electrostatic interactions were computed using the particle-mesh Ewald technique [77,78] with a correction for slab geometries. For equilibrium simulations, all atoms were allowed to move except for those in the outer 0.4 nm of each surface; however, for shear simulations, atoms in the bottom surface were only allowed to move in the *z*-direction (normal to the monolayer plane), with a constant normal force applied to these atoms. Constant normal loads of 5, 15, and 25 nN were considered. To induce shear, atoms in the top surface were allowed to move only in the *x*-direction (the direction of shear) and this surface was coupled via a force constant of 240 kcal/mol/Å2 to a fixed point moving at a velocity of 10 m/s, yielding shear at a constant average velocity. Flow control of simulations and analysis of partially chemisorbed monolayers was achieved using the Signac and Signac Flow packages [79]. The full MoSDeF workflow for these simulations, including initialization, analysis, and run scripts, is hosted on Github and is available at https://github.com/summeraz/xlink-screening.

We note that the simulations of chemisorbed monolayer systems performed at constant separation yield standard deviations in normal load below 5%, so under the conditions of this study, "constant separation" and "constant normal load" ensembles appear to be mostly equivalent.

2. Determination of System Properties

For monolayer systems at equilibrium and undergoing shear, the nematic order parameter (S_2) and average tilt angle (q) were calculated using the following approach. First, average tilt was determined from the characteristic vector, $\hat{\boldsymbol{u}}$, describing the long axes of the alkylsilane monomers; $\hat{\boldsymbol{u}}_k$ for monomer k can be found from the moment of inertia tensor of k, $\boldsymbol{I}_k$, where each component of the 3 × 3 matrix is given by

$$I_{"\#,\%} = \sum_{0)12} m_{)} {*}r_{),}\delta_{\#\%} - r_{)\#} r_{)\%} /. \quad \text{(S1)}$$

N is the total number of atoms in the monomer, m_i is the mass of atom i, $\boldsymbol{r}_i$ is the position of i relative to the molecule's center-of-mass, and dab is the Kronecker delta. $\hat{\boldsymbol{u}}_k$ is the eigenvector associated with the smallest eigenvalue of $\boldsymbol{I}_k$ [80]. Given $\hat{\boldsymbol{u}}$, S_2 can be calculated by constructing the second-rank ordering tensor for each monomer k, $\boldsymbol{Q}_k$, where each element of the 3 × 3 matrix is given by

$$Q_{"\#,\%} = \frac{1}{N} \sum_{i=1}^{N} m_i \left[\frac{3}{2} u_{"\#} u_{"\%} - \delta_{\#\%} 7. \right. \quad \text{(S2)}$$

The director of molecule k, $\hat{\boldsymbol{n}}_k$, is given by the eigenvector associated with the largest eigenvalue of $\boldsymbol{Q}_k$. The order parameter S_2 is

$$S_{,} = \langle \frac{3}{2} \cos^{,} - \frac{1}{2} \rangle$$

where cos qk = $\hat{\boldsymbol{n}}_k \times \hat{\boldsymbol{u}}_k$, and the brackets 'áñ' denote an average over all monomers [81].

The gauche defect fraction was also calculated for monolayers at equilibrium. In alkylsilane monomers, the dihedral angle (ϕ) for twisting a C–C–C–C quadruplet about the central C–C bond is given by

$$\phi = \mathrm{atan2}\left(([\boldsymbol{b}_1 \times \boldsymbol{b}_2] \times [\boldsymbol{b}_2 \times \boldsymbol{b}_3]) \cdot \frac{\boldsymbol{b}_2}{|\boldsymbol{b}_2|}, [\boldsymbol{b}_1 \times \boldsymbol{b}_2] \cdot [\boldsymbol{b}_2 \times \boldsymbol{b}_3]\right) \quad \text{(S3)}$$

where $\boldsymbol{b}_1$, $\boldsymbol{b}_2$, and $\boldsymbol{b}_3$ are vectors representing the three bonds in the quadruplet; $\boldsymbol{b}_2$ corresponds to the central bond [83]. A dihedral angle is considered to be in the trans state if ϕ is between 90° and 270°, while angles outside this range are considered to be gauche defects [82]. The gauche defect fraction is defined by the fraction of total C–C–C–C dihedral angles that are in the gauche state.
